# Atorvastatin improves motor function, anxiety and depression by NOX2-mediated autophagy and oxidative stress in MPTP-lesioned mice

**DOI:** 10.18632/aging.202189

**Published:** 2020-12-03

**Authors:** Junqiang Yan, Jiarui Huang, Anran Liu, Jiannan Wu, Hua Fan, Mengmeng Shen, Xiaoyi Lai, Hongxia Ma, Wenjie Sun, Jianxue Yang, Yunqi Xu

**Affiliations:** 1 Neuromolecular Biology Laboratory, The First Affiliated Hospital, College of Clinical Medicine of Henan University of Science and Technology, Luoyang 471003, P.R. China; 2 Department of Neurology, The First Affiliated Hospital, College of Clinical Medicine of Henan University of Science and Technology, Luoyang 471003, P.R. China; 3 Department of Pharmacy, The First Affiliated Hospital, College of Clinical Medicine of Henan University of Science and Technology, Luoyang 471003, P.R. China; 4 School of Nursing of Henan University of Science and Technology, Luoyang 471003, P.R. China; 5 Department of Neurology, Nanfang Hospital of Southern Medical University, Guangzhou 510515, P.R. China

**Keywords:** Parkinson’s disease, atorvastatin, oxidative stress, autophagy, NADPH oxidase 2

## Abstract

Parkinson’s disease (PD) is a neurodegenerative disease caused by the loss of dopaminergic neurons. It is characterized by static tremors, stiffness, slow movements, and gait disturbances, but it is also accompanied by anxiety and depression. Our previous study showed that atorvastatin could reduce the risk of PD, but the mechanism is still unclear. In this paper, Our findings showed that atorvastatin increased muscle capacity and the coordination of movement and improved anxiety and depression. Atorvastatin could decrease the expression of α-synuclein Ser129 and NADPH oxidase 2 (NOX2), increase the protein expression of LC3II/I, and promote autophagy flow. To further confirm that atorvastatin protection was achieved by inhibiting NOX2, we injected at midbrain with NOX2 shRNA (M) lentivirus and found that silent NOX2 produced the same effect as atorvastatin. Further research found that atorvastatin could reduce MPTP-induced oxidative stress damage, while inhibiting NOX2 decreased the antioxidative stress effect of atorvastatin. Our results suggest that atorvastatin can improve muscle capacity, anxiety and depression by inhibiting NOX2, which may be related to NOX2-mediated oxidative stress and autophagy. Atorvastatin may be identified as a drug that can effectively improve behavioral disorders. NOX2 may be a potential gene target for new drug development in PD.

## INTRODUCTION

Parkinson’s disease (PD) has become increasingly prevalent throughout human life, and the prevalence rate is up to 1.7% in China. [1] PD involves static tremors, stiffness, slow movements, and gait disturbances, and it is also accompanied by depression and anxiety These non-motor symptoms have a dramatic impact on the quality of life of patients. There are still no effective drugs or methods to prevent and treat PD. Therefore, it is particularly urgent to explore the mechanism of dopaminergic (DA) neuron injury and find a drug for prevention and preventive treatment that can prevent or delay PD progression.

Atorvastatin is a hydroxymethylglutaryl-coenzyme A (HMG-CoA) reductase inhibitor that can inhibit the synthesis of endogenous cholesterol and regulate blood lipids. A series of studies have confirmed that statins are effective against PD [2, 3]. Our previous studies have also shown that simvastatin and lovastatin can reduce the inflammatory response and oxidative stress of dopaminergic neurons [4–9], which can reduce the risk of PD. However, it is still unclear how atorvastatin affects PD. Extending the clinical application of atorvastatin to the field of PD is an important challenge for neuroscientists.

Pathology has shown that Lewy bodies may be the main cause of PD. The misfolding and aggregation of α-Syn promotes its extracellular secretion, and it in turn migrates to neighboring neurons and glial cells, promoting the formation of Lewy bodies and accelerating the death of dopamine neurons.

Autophagy regulates the expression of α-Syn and thus influences the progression of PD [10]. In PD cell models, aggregated α-Syn is mainly eliminated by autophagy. Intracellular inhibition of autophagy results in the accumulation and neurodegeneration of intracellular α-Syn [11]. Autophagy degradation of α-Syn can reduce dopaminergic neuron damage. In the SH-SY5Y PD cell model, the natural compound curcumin can reduce the accumulation of α-Syn and induce neuroprotection by inducing autophagy [12]. Autophagy can phagocytose and degrade α-Syn, thereby reducing the damage to dopaminergic neurons [13].

The activation of autophagy has a positive effect on the elimination of misfolded and aggregated proteins and can prevent neurodegeneration. In the A53T transgenic Parkinson’s mouse model (7-8 months), nilotinib (tyrosine kinase inhibitor) can enter the brain tissue and increase the autophagic clearance of α-Syn by upregulating Beclin-1, which protects dopaminergic neurons, thereby improving the motor function of PD mice [14]. Increasing autophagy can reduce the damage to neurons caused by the aggregation of α-Syn. Furthermore, blocking the autophagy pathway can lead to the accumulation of α-Syn and increase neuronal death [15]. In PD models, α-Syn degradation in the brains of PD mice can be increased by stimulating autophagy [16].

In addition to the aggregation of α-Syn, MPTP mouse models also have oxidative stress damage and mitochondrial dysfunction. MPTP dehydrogenates in substantia nigra neurons to generate MPP^+^ and inhibit oxidative phosphorylation by inhibiting mitochondrial complex I, thereby inducing changes in related oxidative stress indicators. Changes in the Keap1/Nrf2 signaling pathway can reduce antioxidant stress damage in the PD model and can change corresponding factors, such as HO-1 and NQO-1. Therefore, we are more concerned about whether atorvastatin can inhibit the effect of NOX2-mediated oxidative stress and autophagy in animal models, thereby protecting neurons.

Our previous study demonstrated that statins, especially atorvastatin, could reduce the risk of PD [9]. However, there is no clear evidence that statins can affect autophagy. In this study, we used an MPTP-lesioned mouse model of PD to study whether atorvastatin could improve behavioral disorders by inhibiting NOX2-mediated oxidative stress and autophagy.

## RESULTS

### Atorvastatin improves depression in MPTP-lesioned mice

**Figure 1:**
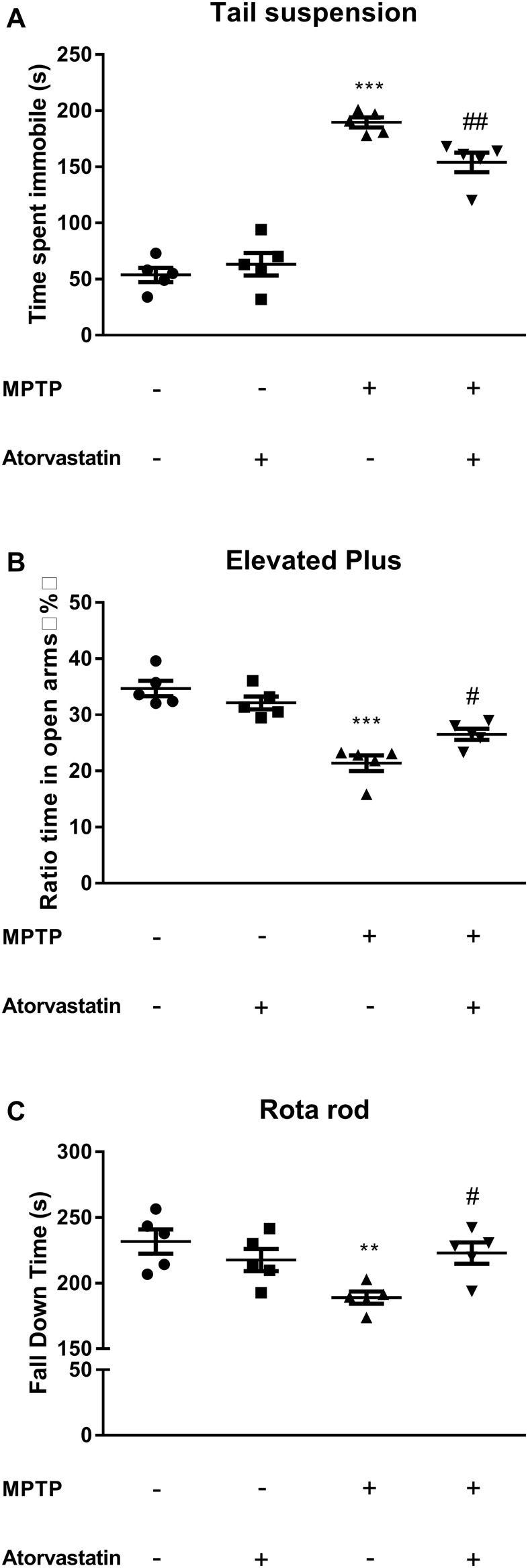
**Effects of atorvastatin on behavior in PD mice.** (**A**) The detection of depression using the tail suspension test. (**B**) The detection of anxiety using the elevated plus maze test. The results were expressed as the percentage of time spent in open arms and the whole experiment time ratio. (**C**) The detection of muscle capacity using the rotarod test. The results were expressed as the fall down time(s) of the whole experiment time. The results compared to the control group are expressed as ^**^p < 0.01, ^***^p < 0.001. The results compared to the MPTP group are expressed as ^#^*p* < 0.05.

The tail suspension experiment tested the depression status of the mice, and the immobility time of the limbs and body reflected depression in the mice. As shown in Figure 1A, the immobility time was longer in the MPTP group than in the control group (F(1,7) = 158.26, *p* < 0.0001). After atorvastatin treatment, the immobility time was significantly reduced compared with that in the MPTP mice (F(1,7) = 15.32, *p* = 0.0185) but was significantly increased compared with that in the control group (F(1,7) = 49.36, *p* = 0.0007).

### Atorvastatin improves anxiety in MPTP-lesioned mice

To verify the effect of atorvastatin on the anxiety of MPTP mice, we tested the anxiety of the mice with the elevated plus maze test. The anxiety level in the open-field test was determined based on the time the mice spent in a corner or on the dark side of the enclosure. The longer they stayed in the open arms, the lower their anxiety. As shown in Figure 1B, the ratio of the time the MPTP mice spent in the center to the time spent in a corner or in darkness within 10 min was significantly greater than the same ratio for mice in the control group, as shown in Figure 1B (F(1,7) = 28.00, *p* = 0.0047). This ratio was significantly reduced in the MPTP mice after atorvastatin treatment (F(1,7) = 9.16, *p* = 0.0236). However, some level of anxiety persisted after treatment with atorvastatin, as this ratio remained greater than that of the control group (F(1,7) = 11.26, *p* = 0.0106).

### Atorvastatin improves muscle capacity in MPTP mice

The classic rotarod test is used to measure the movement capacity of mice. We noticed that atorvastatin significantly improved depression and anxiety in the MPTP mice, so we were interested in whether atorvastatin could improve muscle control. The results of the rotarod test are shown in Figure 1C. The MPTP mice took significantly less time to fall off the rod than the control group (F(1,7) = 19.57, *p* = 0.0067). However, atorvastatin treatment in the MPTP mice significantly increased the length of time taken to fall relative to untreated MPTP mice (F(1,7) = 9.86, *p* = 0.0358). Therefore, it could be inferred that atorvastatin treatment improved muscle capacity in MPTP mice because no other significant differences were seen relative to the control group (F(1,7) = 3.72, *p* = 0.0712).

### Atorvastatin increases the expression of tyrosine hydroxylase and decreases phosphorylated Ser129 in MPTP-lesioned mice

**Figure 2:**
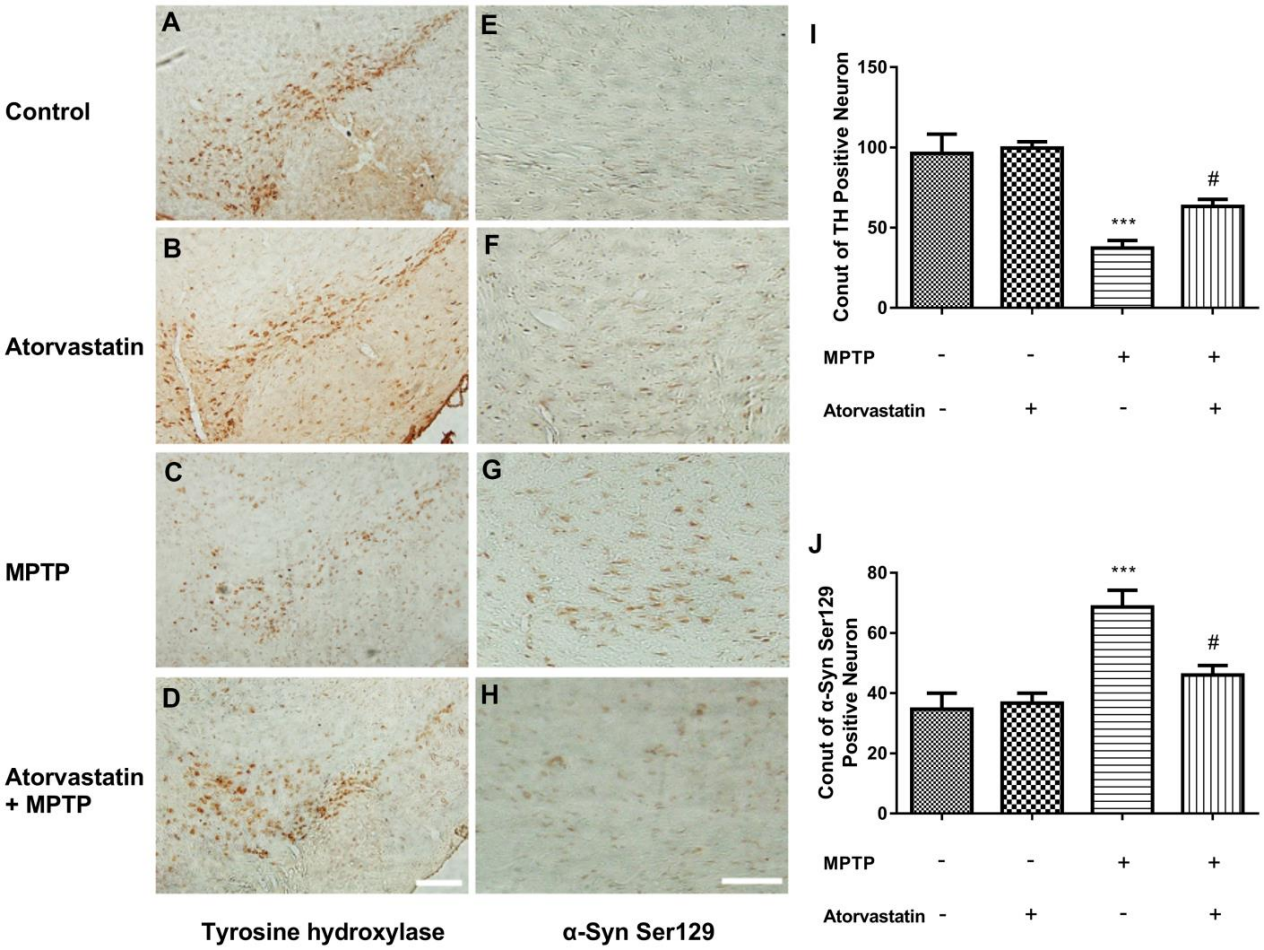
**Atorvastatin reduces the number of dead MPTP-induced nigral neurons in the autophagic flux marker PD mouse.** Immunohistochemistry of tyrosine hydroxylase in the substantia nigra neurons of an autophagic flux marker mouse model (**A**–**D**). Immunohistochemistry of α-Syn Ser129 substantia nigra neurons in an autophagic flux marker mouse model (**E**–**H**). Bar = 100 μm. Quantitative analysis of TH positive neurons and α-syn ser129 positive neurons (**I**, **J**). The results are expressed as ***p < 0.001 compared to atorvastatin/MPTP (-/-). The results were expressed as ^#^*p* < 0.05 compared to atorvastatin/MPTP (-/+).

Tyrosine hydroxylase (TH) was significantly reduced in MPTP-lesioned mice, but after the administration of atorvastatin, TH significantly increased compared to the MPTP group, as shown in Figure 2A–2D, 2I. Furthermore, we also measured the phosphorylation of α-Syn at Ser129, as shown in Figure 2E–2H, 2J. After MPTP lesioning, phosphorylated Ser129 was increased in the substantia nigra, as shown in Figure 2G. After atorvastatin treatment, the number and extent of phosphorylated Ser129 were significantly decreased compared to the MPTP group.

### Atorvastatin promotes autophagic flux in MPTP-lesioned mCherry-eGFP tag mice

**Figure 3:**
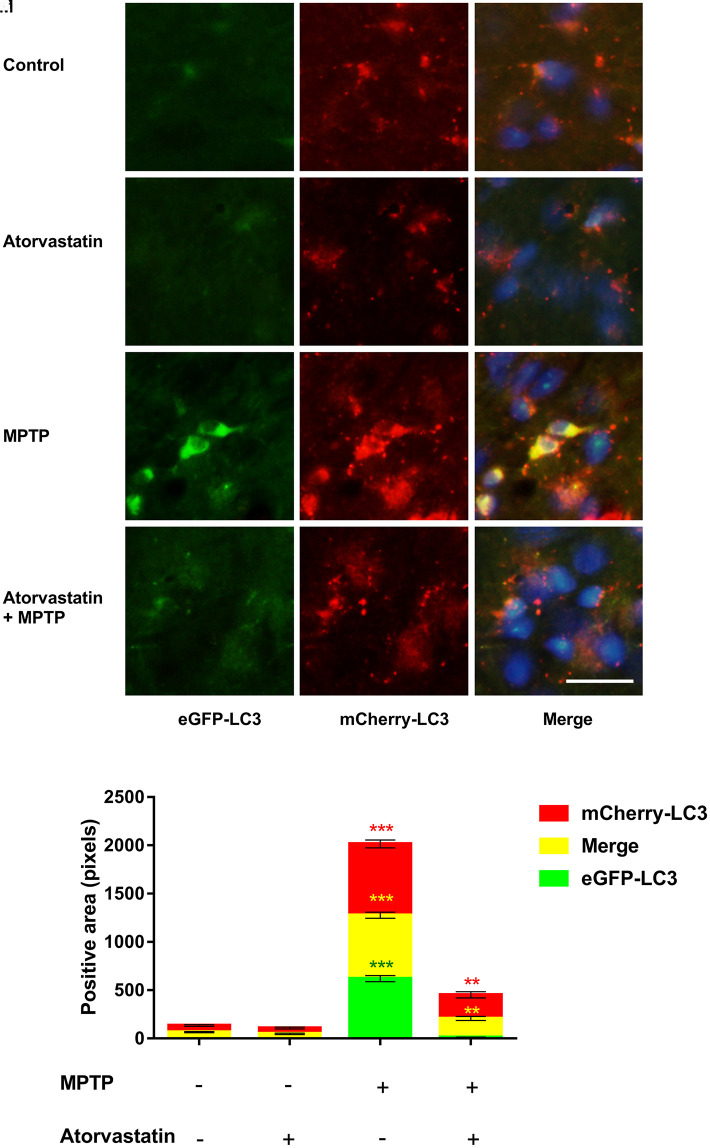
**Changes in autophagic flux in MPTP mice.** The expression of eGFP-LC3 in substantia nigra neurons of autophagic flux marker mouse models(**A**–**D**). The expression of mCherry-LC3 in substantia nigra neurons of autophagic flux marker mouse models(**E**–**H**). The merged figures show the expression of eGFP-mCherry-LC3 (**I**–**L**). Bar = 20 μm. Quantitative analysis of eGFP, mCherry and merged positive area (**M**). The results are expressed as ***p < 0.001 compared to atorvastatin/MPTP (-/-). The results were expressed as **p < 0.01 compared to atorvastatin/MPTP (-/+).

The fluorescence labeling of eGFP-mCherry-LC3 can reflect changes in the autophagic flux of dopaminergic neurons. We detected changes in autophagic flux in the substantia nigra by immunofluorescence, as shown in Figure 3A–3M. Compared to the control group, after atorvastatin treatment, the fluorescence intensity of eGFP and mCherry in the substantia nigra did not significantly change. In MPTP-lesioned mCherry-eGFP-tagged mice, the fluorescence of eGFP and mCherry significantly increased and tended to aggregate from the dispersion state, similar to small particles, to form a group, as shown in Figure 3G. Atorvastatin decreased the eGFP fluorescence intensity compared to MPTP, as shown in Figure 3D. The yellow color after the merge tended to be approximately red in Figure 3K.

### Atorvastatin decreases the expression level of α-Syn Ser129 and increases the ratio of LC3II/LC3I in MPTP-lesioned mice

**Figure 4:**
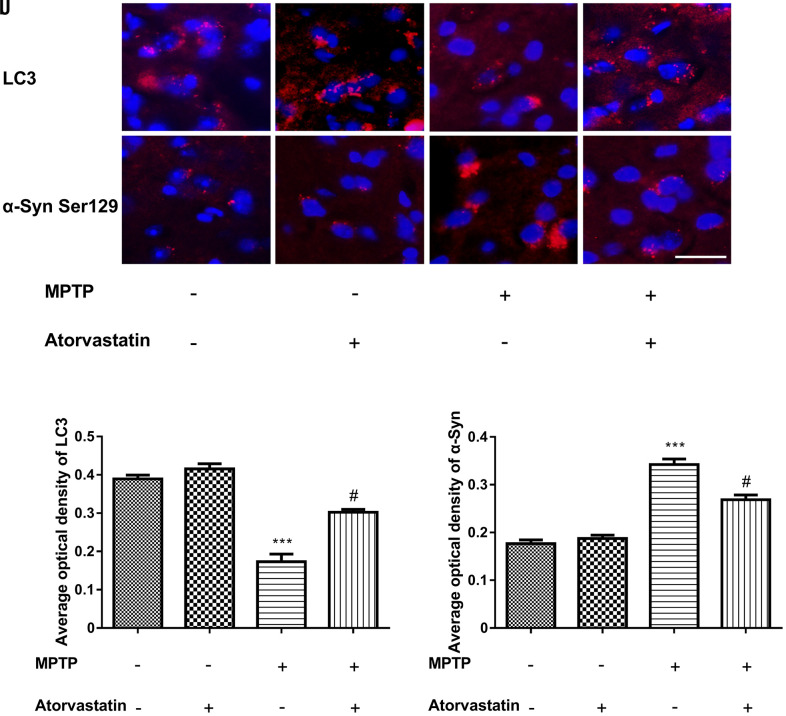
**Changes in the expression of LC3 and α-syn Ser129 in MPTP mice.** The expression of LC3 in the substantia nigra neurons of C57BL/6 mice (**A**–**D**). The expression of α-Syn Ser129 in the substantia nigra neurons of C57BL/6 mice (**E**–**H**). Bar = 20 μm. Quantitative analysis of LC3 and α-Syn average optical (**I**, **J**). The results are expressed as ***p < 0.001 compared to atorvastatin/MPTP (-/-). The results were expressed as ^#^*p* < 0.05 compared to atorvastatin/MPTP (-/+).

The immunofluorescence results of the expression of LC3 and α-Syn Ser129 are shown in Figure 4A–4J. Atorvastatin alone had no significant effect on LC3 expression compared with the control group, while MPTP treatment significantly decreased the level of LC3 aggregation. Atorvastatin reversed the decrease in LC3 caused by MPTP damage in MPTP-lesioned mice. We also found that atorvastatin alone had no significant effect on α-Syn Ser129, and MPTP significantly increased the expression level of α-Syn Ser129. The increased α-Syn Ser129 could be reversed by atorvastatin in MPTP-lesioned mice.

**Figure 5:**
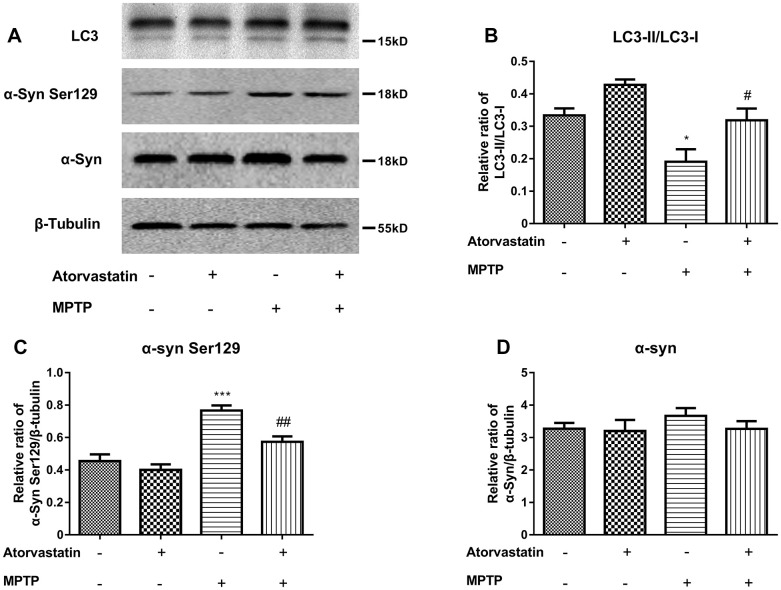
Western blot results show the protein expression (**A**) of LC3-II/LC3-I (**B**), α-Syn Ser129 (**C**) and α-Syn (**D**) in the substantia nigra neurons of C57BL/6 mice. The results compared to atorvastatin/MPTP (-/-) are expressed as ^*^p < 0.05, ^***^p < 0.001. The results compared to atorvastatin/MPTP (-/+) were expressed as ^#^*p* < 0.05 or ^##^*p* < 0.01.

To further confirm our conclusions, we analyzed the protein expression of LC3 and α-Syn Ser129, as shown in Figure 5A–5D. Atorvastatin alone had no significant effect on LC3 or α-Syn Ser129 compared with the control group. MPTP decreased the ratio of LC3II/LC3I Figure 5A, 5B) and increased the expression level of α-Syn Ser129 (Figure 5A, 5C) compared to the control group. Atorvastatin decreased the expression level of α-Syn Ser129 and increased the ratio of LC3II/LC3I compared to the MPTP-lesioned group.

### Effect of atorvastatin on the protein expression of NOX2, LC3II/LC3I and α-Syn Ser129 in MPTP-lesioned mCherry-eGFP-tagged mice

**Figure 6:**
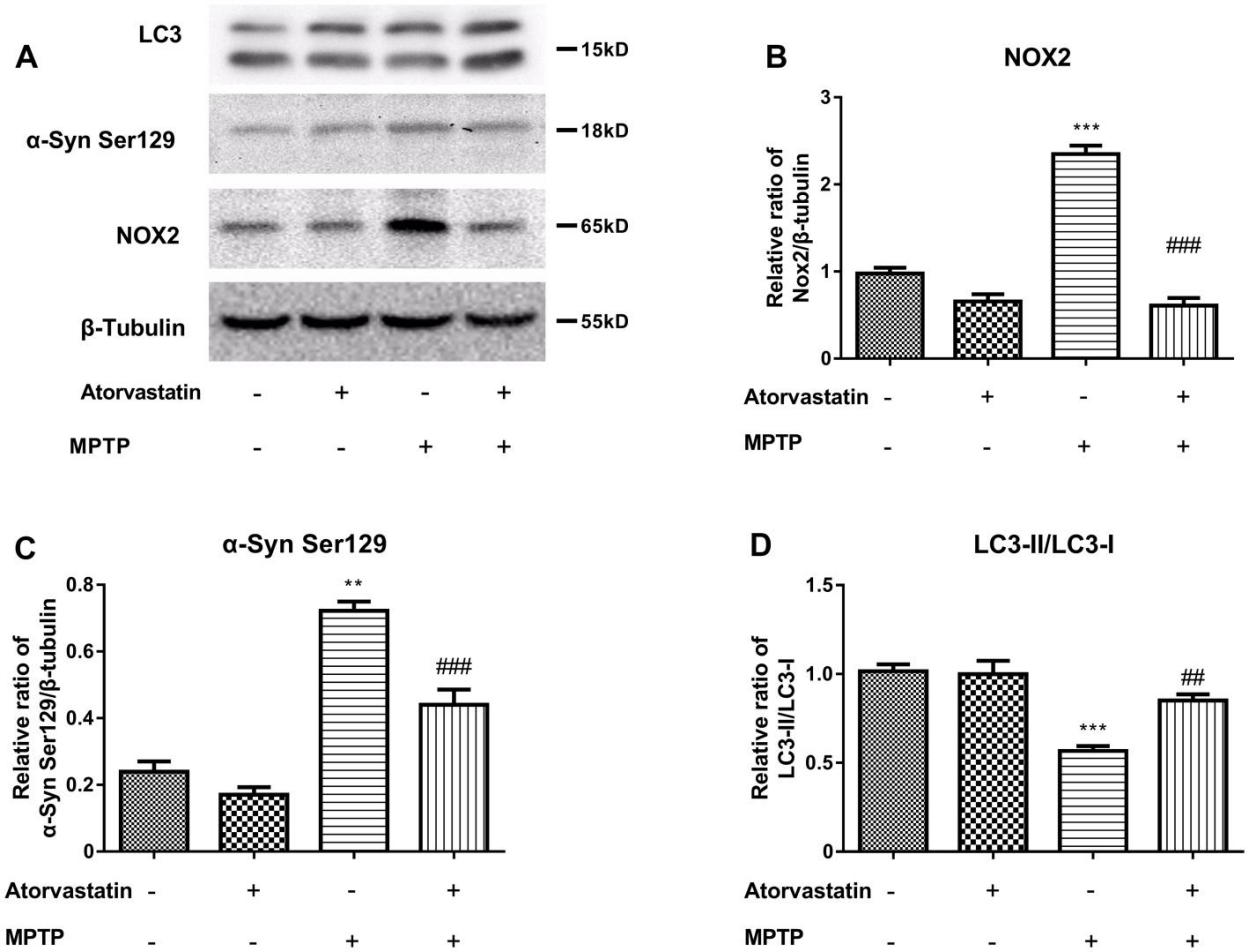
****The α-syn Ser129, LC3 and CYBB/NOX2 changed in the MPTP-induced autophagic flux mice (**A**–**D**). The western blot results show the protein expression of NOX2 (**B**), α-Syn Ser129 (**C**) and LC3-II/LC3-I (**D**) in the substantia nigra neurons of autophagic flux mice. The results compared to atorvastatin/MPTP (-/-) are expressed as ^*^p < 0.05, ^**^p < 0.01, ^***^p < 0.001. The results/MPTP (-/+) were expressed as ^##^*p* < 0.01 or ^###^*p* < 0.001 compared to atorvastatin.

To further study the protective mechanism of atorvastatin in PD mice, we used MPTP-injured mCherry-eGFP-tagged mice as an animal model of PD. Compared with the control group, the atorvastatin group had no significant effect on NOX2, LC3 or α-Syn Ser129. MPTP increased the protein expression of NOX2 (Figure 6B) and α-Syn Ser129 (Figure 6C) and decreased the ratio of LC3II/LC3I (Figure 6D) compared to the control group. Compared to the MPTP group, atorvastatin decreased the protein expression of NOX2 and α-Syn Ser129 and increased the ratio of LC3II/LC3I.

### Inhibition of NOX2 increases the protein level of LC3 and decreases α-Syn Ser129 in MPTP-lesioned mCherry-eGFP-tagged mice

**Figure 7:**
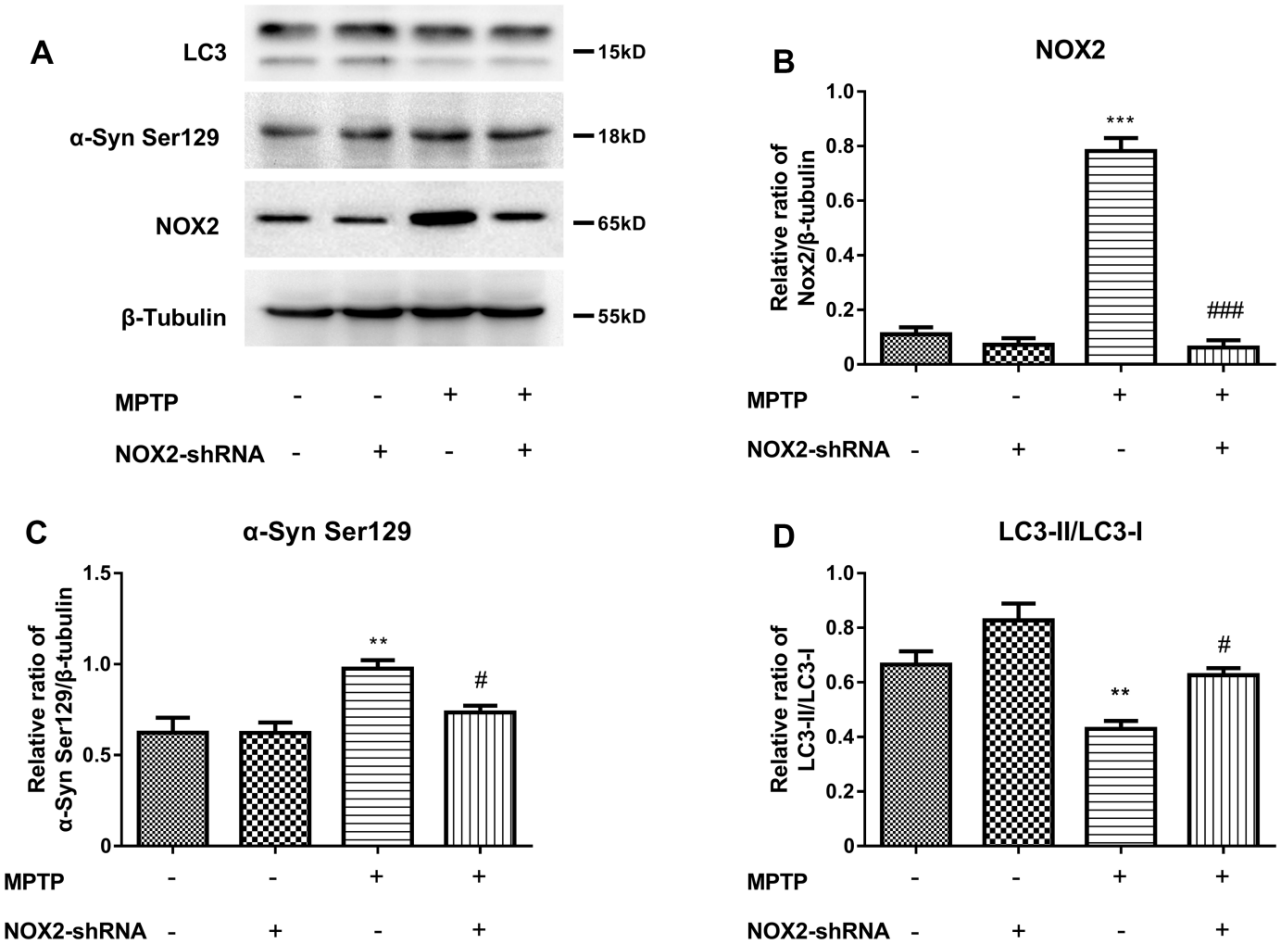
Inhibition of NOX2 increases the protein level of LC3 and decreases α-Syn Ser129 in MPTP-lesioned mCherry-eGFP-tagged mice (**A**–**D**). The western blot results showed the protein expression of NOX2 (**B**), α-Syn Ser129 (**C**) and LC3-II/LC3-I (**D**) in the substantia nigra neurons of C57BL/6 mice. The results are expressed as ^*^p < 0.05 or ^**^p < 0.01 compared to NOX2-shRNA/MPTP (-/-). The results were expressed as ^#^*p* < 0.05 or ^###^*p* < 0.001 compared to NOX2-shRNA/MPTP (-/+).

To further explore the relationship among NOX2, LC3 and α-Syn Ser129, we used gp91-phox shRNA (m) lentivirus to perform a brain localization injection in mCherry-eGFP tagged mice to inhibit the expression of NOX2. The protein expression of NOX2, LC3 and α-Syn Ser129 in the substantia nigra after inhibiting NOX2 is shown in Figure 7A–7D. Figure 7B shows that the expression of NOX2 was significantly inhibited. The changes in α-Syn Ser129 and LC-3II/LC3-I were similar to those with atorvastatin treatment after NOX2 was inhibited. As shown in Figure 7C, compared to the control group, MPTP significantly increased the protein expression of α-Syn Ser129, while inhibiting NOX2 reversed the increase in α-Syn Ser129 caused by MPTP. Compared to the MPTP group, after NOX2 was inhibited, the significant increase in the ratio of LC3-II/LC3-I was inhibited.

### Inhibition of NOX2 promotes autophagic flux in MPTP-lesioned mCherry-eGFP-tagged mice

**Figure 8:**
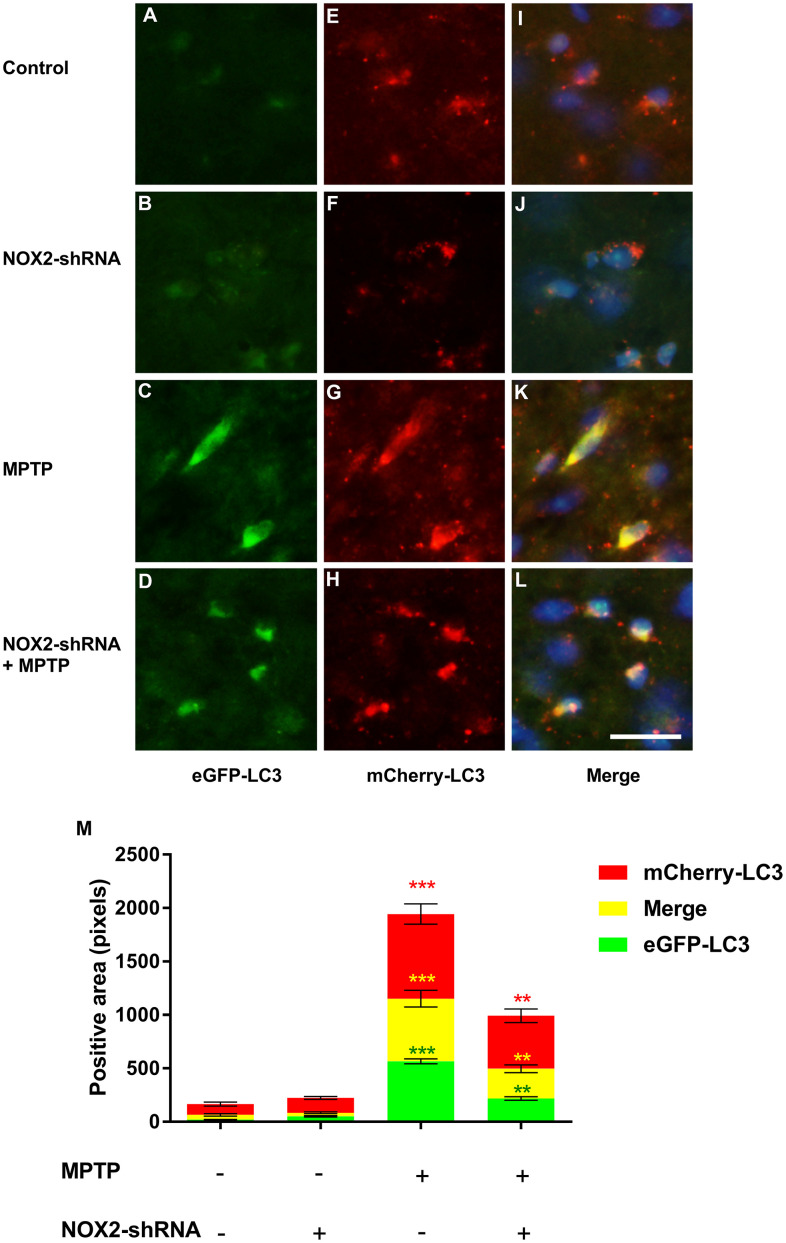
**The effect of NOX2 inhibition on autophagic flux.** The expression of eGFP-LC3 in the substantia nigra neurons of the marker mouse model (**A**–**D**). The expression of mCherry-LC3 in the substantia nigra neurons of the autophagic flux marker mouse model (**E**–**H**). The merged figures show the expression of eGFP-mCherry-LC3 (**I**–**L**). Bar = 20 μm. Quantitative analysis of eGFP, mCherry and merged positive area (**M**). The results are expressed as ***p < 0.001 compared to atorvastatin/MPTP (-/-). The results were expressed as **p < 0.01 compared to atorvastatin/MPTP (-/+).

To further explore the effect of NOX2 on autophagic flux, we used gp91-phox shRNA (m) lentivirus to perform a brain localization injection in mCherry-eGFP-tagged mice to inhibit the expression of NOX2. The changes in autophagic flux in the substantia nigra after inhibiting NOX2 are shown in Figure 8A–8M. The role of NOX2-shRNA was similar to that of atorvastatin. Compared to the control group, after atorvastatin treatment, the fluorescence intensity of eGFP and mCherry in the substantia nigra did not significantly change. In MPTP-lesioned mCherry-eGFP-tagged mice, the fluorescence of eGFP and mCherry significantly increased and tended to aggregate from the dispersion state. Inhibiting NOX2 decreased the eGFP fluorescence intensity compared to the MPTP group, and the yellow color after the merge tended to be approximately red.

### Atorvastatin increases antioxidant stress by inhibiting NOX2

**Figure 9:**
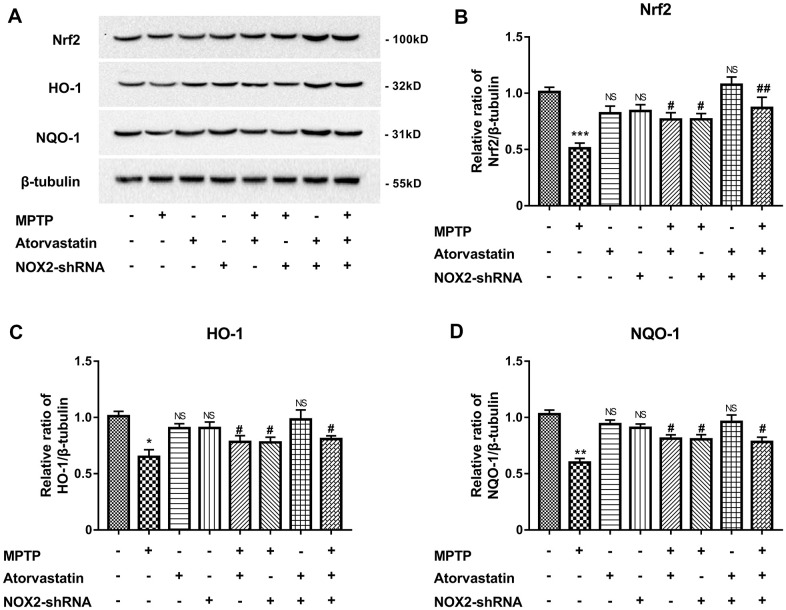
****Atorvastatin rescues the expression of antioxidant stress Nrf2, HO-1 and NQO-1 (**A**–**D**) by inhibiting NOX2 in MPTP mice. The results are expressed as ^*^p < 0.05 or ^**^p < 0.01 or ^***^p < 0.001; NS indicates no significance compared to MPTP/atorvastatin/NOX2-shRNA (-/-/-). The results are expressed as ^#^*p* < 0.05 or ^##^*p* < 0.05 compared to MPTP/atorvastatin/NOX2-shRNA (+/-/-).

MPTP can induce oxidative stress by the Nrf2/Keap1/ARE pathway. Therefore, we explored the effect of atorvastatin on the antioxidant stress of Nrf2 and downstream HO-1 and NQO-1. As shown in Figure 9A, 9B, Nrf2 expression was significantly reduced under MPTP induction, and the expression of single NOX2-shRNA or atorvastatin treatment on Nrf2 was not significant. Atorvastatin and NOX2 inhibition significantly increased Nrf2 expression in the MPTP-induced group compared with the single MPTP group. As shown in Figure 9A, 9C and 9D, the expression of HO-1 and NQO-1 was similar to that of Nrf2. MPTP significantly reduced the expression of HO-1 and NQO-1. The inhibition of atorvastatin and NOX2 could rescue the expression levels of HO-1 and NQO-1. It can be concluded that atorvastatin can increase the level of antioxidant stress by inhibiting NOX2, affecting Nrf2 and downstream HO-1 and NQO-1.

## DISCUSSION

MPTP is a highly fat-soluble drug that can quickly pass through the cell membrane after intraperitoneal injection; it quickly enters the brain and is converted into MPP^+^. After MPP^+^ enters DA neurons, the mitochondrial respiration chain is destroyed, and the cell's energy supply is interrupted, which damages DA neurons and produces clinical symptoms of PD. In this study, we used wild-type MPTP-injured mice and double-fluorescently labeled MPTP autophagy mice as animal models of Parkinson's disease to explore the effect of atorvastatin on behavioral stress, oxidative stress and autophagy.

Regarding the underlying mechanism of Parkinson's disease in terms of the treatment method, the modulation of autophagy is very promising because strong evidence indicates that autophagy impairment can lead to Parkinson’s disease [17–19]. Previous evidence has shown that autophagy can increase the degradation of synuclein through proteasome and autophagic lysosome pathways [10]. The MPTP model had a significant effect on the damage of dopaminergic neurons in the substantia nigra. While common antioxidants can reduce oxidative stress damage, they cannot eliminate the increase in neuronal phosphorylated synuclein. Our findings showed that atorvastatin could increase the survival rate of tyrosine hydroxylase neurons and LC3 levels and decrease the protein expression of α-synuclein, which shows that atorvastatin's protective effect on PD mice may be related to LC3-mediated autophagy because enhanced autophagy can degrade α-synuclein and protect dopaminergic neurons. Our results are consistent with those of a study in which deletion of the autophagy-related gene ATG5 could impair motor and cognitive function as well as reduce tyrosine hydroxylase neurons and dopamine levels in the striatum of mice [19].

The measurement of autophagy biomarkers cannot directly reflect the dynamic changes in autophagy, while autophagic flux may be a better choice. To further confirm our speculations, we used mCherry-eGFP-tagged mice instead of wild-type mice in the experiment as a PD model. Changes in fluorescence can directly indicate the changes in autophagosome formation and degradation after binding to lysosomes in mCherry-eGFP-tagged mice. Fluorescently labeled LC3 does not easily disappear in the acidic state of autophagosomes, while eGFP does the opposite. Therefore, changes in red and green fluorescence can directly reflect changes in autophagic flux. We found that autophagic flux was significantly inhibited in PD mice, and atorvastatin could promote autophagic flux. Autophagy can promote the degradation of α-synuclein by the proteasome and autophagolysosomal pathways [10]. We speculated that atorvastatin may increase the degradation of α-synuclein by promoting autophagy, thereby improving depression, anxiety and muscle capacity.

Interestingly, atorvastatin reduced the expression of NOX2 while increasing the LC3 expression level in our study. To confirm the relationship between PD and NOX2, we inhibited NOX2 expression by NOX2 shRNA and found that inhibition of NOX2 produced the same results as atorvastatin, which were improvements in anxiety, depression, and exercise capacity. Further study found that inhibition of NOX2 could increase the ratio of LC3II/LC3I and promote autophagic flux. The genetic downregulation of NOX2 could restore the inhibition of autophagic flux induced by MPTP, increase LC3 levels, and decrease the protein expression of α-synuclein. Our results are consistent with those of a study in which MPTP led to upregulation of NOX2, which can increase the expression of reactive oxygen species (ROS). NOX2-derived ROS can stimulate the AKT-mTOR pathway and cause autophagy inhibition [20]. Jaishy’s study also showed that Nox2 activation generated a large number of ROS that inhibit lysosomal function and led to autophagy impairment [21], which is consistent with our research. Moreover, our study on the effect of atorvastatin on antioxidative stress confirmed that atorvastatin can exert a positive effect on Nrf2 and downstream HO-1 and NQO-1 by inhibiting NOX2 and increasing the tolerance of neurons to oxidative stress damage from MPTP.

A previous study showed that activation of autophagy by rapamycin significantly reduced dopaminergic neuronal death mediated by paraquat exposure in vivo and improved the impairment of motor and cognitive functions [22]. Our study shows improved not only muscle function but also anxiety and depression, suggesting a protective role of atorvastatin. These results agree with the prophylactic effect of atorvastatin observed on emotional and motor impairments in MPTP-lesioned rats [23].

## CONCLUSIONS

Our data suggest that MPTP can inhibit autophagy and increase α-synuclein, while enhanced autophagy by inhibiting NOX2 can decrease α-synuclein to protect dopaminergic neurons. Atorvastatin can activate autophagy by inhibiting NOX2 in PD mice, which may provide substantial benefits for PD animal models. Atorvastatin may be identified as a drug that can effectively target NOX2 and improve the clinical symptoms of Parkinson's disease. NOX2 may be a potential target for the clinical treatment of PD.

## MATERIALS AND METHODS

### PD mouse model

This study was approved by the Ethics Committee/Institutional Review Board of the First Affiliated Hospital of Henan University of Science and Technology. All animals were treated in accordance with the guidelines of the NIH’s Guide for the Care and Use of Laboratory Animals and followed the guidelines of the International Association for the Study of Pain (IASP). A MPTP mouse model was used, with 6-8-week-old male C57BL/6 mice from HFK Co., Ltd (Beijing, China). These mice underwent intraperitoneal injection of MPTP (30 mg/kg) from Sigma-Aldrich (M8096, St. Louis, MO, USA) once a day for 5 consecutive days. At the same time, atorvastatin (10 mg/kg/day) was administered for 5 days, and the control group underwent intraperitoneal injection of saline solution. Under constant temperature and humidity conditions, breeding was carried out under a light/dark cycle of 12/12 hours.

### Stereotactic brain injection

Autophagic flux mice (6-8-week-old male C57BL/6 mice) were prepared with an LC3 lentiviral expression plasmid tagged with an mCherry-eGFP tag. The tagged LC3 lentiviral expression plasmid was injected into the substantia nigra by stereotactic injection. Chloral hydrate was given in the nesteia before the operation. The anesthetized mice were fastened to the craniocerebral locator. Their scalp was cut and exposed to hydrogen peroxide to expose the bone seam. The drill was used to create the needle position (bregma -2.92 mm, lateral 1.50 mm, depth 4.5 mm). The needle was placed in the small hole and retained for 10 minutes after the injection. Close attention was paid to observing the behavioral state of the mice until they were awake, when they were sutured and disinfected.

### Rotarod test

At the beginning of each experiment, the mice were placed in separate isolated lanes. The instrument speed was initially set at 4 rpm, with the first acceleration to 40 rpm after 300 s, after which this speed was maintained for an additional 300 s [24]. The average number of seconds that mice maintained their position on the rod in each experiment was recorded.

### Tail suspension test

The tail-suspension test was used to evaluate depressive-like behaviors in animals. This method is based on the observation that mice suspended by the tail show immobility, which reflects despair. The mice were suspended by the tail with tape on a platform 50 cm above the ground and considered immobile when they showed no physical movement and were passively suspended. Then, limb and body movements were recorded for 5 min [25].

### Elevated plus maze test

A circular swimming pool 120 cm in diameter and 35 cm in height with a constant temperature of 25° C was divided into four equal areas. A 10-cm-diameter circular platform was placed in the middle of each area, with the platform submerged just below the surface of the water. The mice were trained for 1 week before the experiment. During the training, the mice were placed on the platform for 2 min to become familiarized with the environment around the platform. The mice were then placed at the edge of the pool, opposite the platform, and were allowed to swim for 3 min to find the platform. When the mouse did not find the platform within 3 min, the mouse was directed to the platform and kept there for 2 min.

### Immunohistochemistry

The brain sections were incubated with methanol and hydrogen (9:1) peroxide. PBS was used to wash them and block them with goat serum, and then they were incubated overnight with a primary antibody mixed solution of 1% BSA. They were incubated for 2 hours with HRP containing secondary antibodies mixed with 1% BSA. The DAB method was used for dyeing, and then dimethylbenzene was dehydrated and sealed with gum. The following primary antibodies were used: TH antibody (Novus Cat# NB300-109, RRID:AB_10077691) and phospho-α-Syn Ser129 antibody (Cell Signaling Technology Cat# 23706, RRID:AB_2798868).

### Immunoblotting

We isolated the substantia nigra of the brain. RAPI lysate was used, the tissue was ground, and the supernatant was extracted for protein quantification. A sample with 20 μg total protein was subjected to SDS-PAGE electrophoresis. It was then transferred using a PVDF membrane and blocked with 5% skim milk. It was incubated overnight at 4° C with a mixture of primary antibodies to 0.5% skim milk TBST solution. It was then washed three times using TBST, incubated with a secondary antibody for 1 hour, and washed three times with TBST. Protein chemiluminescence was detected using ChemiDoc™ (Bio-Rad) and the ECL method.

The following primary antibodies were used: NOX2 antibody (Abcam Cat# ab129068, RRID:AB_11144496), LC3 antibody (MBL International Cat# PM036, RRID:AB_2274121), Nrf2 antibody (Santa Cruz Biotechnology Cat# sc-365949, RRID:AB_10917561), HO-1 antibody (Santa Cruz Biotechnology Cat# sc-136960, RRID:AB_2011613), NQO-1 antibody (Santa Cruz Biotechnology Cat# sc-32793, RRID:AB_628036), phospho-α-Syn Ser129 antibody (Cell Signaling Technology Cat# 23706, RRID:AB_2798868), α-Syn antibody (Cell Signaling Technology Cat# 4179, RRID:AB_1904156), and β-tubulin antibody (Cat# Cat# 1798868).

### Immunohistochemistry

The basic procedure was performed by immunohistochemistry. After incubating the primary antibody, the specific fluorescent-labeled secondary antibody was used for incubation for 2 hours. After washing, DAPI was used for counterstaining. Glycerin was used to mount and observe it under a fluorescence microscope. The following primary antibodies were used: LC3 antibody (MBL International Cat# PM036, RRID:AB_2274121) and α-Syn Ser129 antibody (Cell Signaling Technology Cat# 4179, RRID:AB_1904156).

### Statistical analysis

The data are expressed as the means ± standard errors of the means. The results of the optical density assays and the immunoblotting quantifications of different proteins were analyzed using one-way analysis of variance, followed by Tukey’s post hoc analysis (GraphPad Prism 7.0, USA). *P* values of < 0.05 were regarded as statistically significant.
